# LncRNA *Jpx* induces *Xist* expression in mice using both *trans* and *cis* mechanisms

**DOI:** 10.1371/journal.pgen.1007378

**Published:** 2018-05-07

**Authors:** Sarah Carmona, Benjamin Lin, Tristan Chou, Katti Arroyo, Sha Sun

**Affiliations:** Department of Developmental and Cell Biology, Ayala School of Biological Sciences, University of California, Irvine, Irvine, CA, United States of America; Stanford University School of Medicine, UNITED STATES

## Abstract

Mammalian X chromosome dosage compensation balances X-linked gene products between sexes and is coordinated by the long noncoding RNA (lncRNA) *Xist*. Multiple *cis* and *trans*-acting factors modulate *Xist* expression; however, the primary competence factor responsible for activating *Xist* remains a subject of dispute. The lncRNA *Jpx* is a proposed competence factor, yet it remains unknown if *Jpx* is sufficient to activate *Xist* expression in mice. Here, we utilize a novel transgenic mouse system to demonstrate a dose-dependent relationship between *Jpx* copy number and ensuing *Jpx* and *Xist* expression. By localizing transcripts of *Jpx* and *Xist* using RNA Fluorescence *in situ* Hybridization (FISH) in mouse embryonic cells, we provide evidence of *Jpx* acting in both *trans* and *cis* to activate *Xist*. Our data contribute functional and mechanistic insight for lncRNA activity in mice, and argue that *Jpx* is a competence factor for *Xist* activation *in vivo*.

## Author summary

Long noncoding RNA (lncRNA) have been identified in all eukaryotes but mechanisms of lncRNA function remain challenging to study *in vivo*. A classic model of lncRNA function and mechanism is X-Chromosome Inactivation (XCI): an essential process which balances X-linked gene expression between male and female mammals. The “master regulator” of XCI is lncRNA *Xist*, which is responsible for silencing one of the two X chromosomes in females. Another lncRNA, *Jpx*, has been proposed to activate *Xist* gene expression in mouse embryonic stem cells; however, no mouse models exist to address *Jpx* function *in vivo*. In this study, we developed a novel transgenic mouse system to demonstrate the regulatory mechanisms of lncRNA *Jpx*. We observed a dose-dependent relationship between *Jpx* copy number and *Xist* expression in transgenic mice, suggesting that *Jpx* is sufficient to activate *Xist* expression *in vivo*. In addition, we analyzed *Jpx*’s allelic origin and have provided evidence for *Jpx* inducing *Xist* transcription using both *trans* and *cis* mechanisms. Our work provides a framework for lncRNA functional studies in mice, which will help us understand how lncRNA regulate eukaryotic gene expression.

## Introduction

Mammalian gender is determined by a pair of sex chromosomes (females are XX while males are XY), leading to an inherent imbalance of X-linked gene products between the sexes. Gene dosage is compensated by X-Chromosome Inactivation (XCI), a process which transcriptionally silences one X chromosome in females during early embryonic development [1]. XCI is primarily carried out by a cluster of long noncoding RNA (lncRNA) located on the X chromosome in a region known as the X-inactivation center (Xic) [2,3]. The master regulator of XCI is the lncRNA *Xist*, which coordinates X-linked gene silencing by spreading across the future inactive X (X_i_) [4,5].

How *Xist* is selectively activated in female individuals, but not in males, remains an essential, unresolved question in the field. A “Two Factors Model” has been used to describe XCI initiation and *Xist* regulation, in which competence factors trigger XCI on X_i_ while blocking factors prevent XCI on the active X (X_a_) [6–8]. To determine the number of XCI events, the cell counts the number of X chromosomes relative to autosomes–the X:A ratio [9]. Male cells (X:A = 1:2) typically do not induce XCI while female cells (X:A = 2:2) normally induce one XCI event. When the X:A ratio is disturbed, for example in genetic aneuploidies such as a male XXY (X:A = 2:2), the male cell initiates an XCI event to maintain proper X chromosome dosage [9]. Chromosome counting involves a genetic component, as the X chromosome count and subsequent XCI events must be influenced by a *trans*-diffusible factor [8,10]. A Self-Enhanced Transport (SET) model has also been proposed to describe XCI activation, in which *Xist* exhibits an ultrasensitive (switch-like) response to a competence factor followed by a self-enhanced positive feedback mechanism to maintain *Xist* expression at the initiation of XCI [11].

While the primary *Xist* activating factor is under debate, two competence factors have been described. One candidate is E3-Ubiquitin ligase RNF12 (also known as RLIM), which activates *Xist* expression by targeting and degrading the *Xist* blocking-factor REX1 [12]. In mouse embryonic stem cell (mESC) models that recapitulate XCI during embryonic development, *Rnf12* expression correlates with downregulation of the pluripotency factor NANOG and subsequent *Xist* activation [13]. However, deleting *Rnf12* from mESCs does not prevent XCI from occurring. In one study, a heterozygous *Rnf12* deletion reduced the rate of XCI initiation, but *Xist* RNA clouds were still detected in differentiating mESCs [14]. A later study achieved a homozygous *Rnf12* deletion yet still detected sporadic *Xist* expression from mESCs [13]. Further, when *Rnf12* was conditionally deleted from mouse embryos, no effect on *Xist* expression or XCI was observed [15]. In mice, RNF12 has since been shown to control *Xist* activation during imprinted XCI, a form of XCI in extraembryonic tissues which does not involve the same X-chromosome counting process as random XCI in the embryo [15,16]. A most recent study characterizing *Rnf12* and XCI in mouse extraembryonic tissues and embryo proper revealed *Rnf12* downregulation prior to random XCI in the embryo. [17]. Together, these studies suggest that additional X-encoded factors can activate *Xist* expression and initiate XCI in mESCs and in the mouse embryo.

Another proposed *Xist* activator is *Jpx*, a functional lncRNA whose gene is located just proximal to *Xist*. *Jpx* escapes inactivation and has been found to activate *Xist* expression by binding to and removing CTCF protein from the *Xist* promoter [8,18]. *Jpx* appears necessary for XCI in a mESC model, in which a heterozygous *Jpx* deletion compromised the overall *Jpx* and *Xist* expression and led to lethality in differentiating female ES cells [18]. Intriguingly, the surviving female cells maintained *Xist* induction preferentially associated with the remaining *Jpx* allele in *cis* (on the same chromosome). Importantly, a transgene containing *Jpx*, Tg(Jpx), was able to restore *Xist* expression and rescue the female cell viability, supporting a *trans*-acting role of *Jpx* for *Xist* activation in mESCs [18]. Using the mESC system, another study reported a large deletion of a 500kb genomic region upstream of *Xist*, which includes both *Jpx* and *Rnf12* but nevertheless caused no major defects to the cells [10]. Interestingly, the overall *Xist* expression was significantly decreased in the heterozygous Δ(*Jpx-Rnf12*) differentiating mES cells compared to the cells with heterozygous deletion of *Rnf12* by itself. A transgene containing *Rnf12* was able to rescue the *Xist* expression in these heterozygous Δ(*Jpx-Rnf12*) cells but only up to ~65% of the wildtype level [10]. These observations suggest that *Jpx* is needed for full activation of *Xist* in mESCs.

Further, when the Tg(Jpx) transgene was inserted into wildtype mESCs, *Xist* was ectopically expressed in both male and female cells, indicating that *Jpx* itself is capable of activating *Xist* in *trans* [8]. However, a different transgene (containing *Jpx* and *Ftx*) failed to induce *Xist* expression in mESCs [10,19], although *Jpx* expression levels in these cells were not noted. The current debate on an active role of *Jpx* in XCI, and the effects of transgenic *Jpx* on *Xist* expression in mESCs, prompts the establishment and characterization of *Jpx* transgenic mouse models. In this study, we provide findings from novel transgenic mice to elucidate the relationship between *Jpx* and *Xist* activities *in vivo*.

Specifically, to test if *Jpx* is a competence factor for *Xist* activation in mice, we asked if additional copies of *Jpx* would induce *Xist* expression *in vivo*. We further questioned what genetic mechanism *Jpx* acts through: a *trans* (distal) or *cis* (local) regulatory control. We utilized a pair of overlapping transgene constructs to develop a novel transgenic mouse model, in which we monitored the *Xist* response to *Jpx* expression from a *trans* (on a different chromosome) or *cis* (on the same chromosome or within the transgene) origin. By characterizing phenotypic consequences of the transgenes, particularly expression patterns of *Jpx* and *Xist* in mouse embryonic cells, we determined the function and genetic mechanism of *Jpx* on *Xist* activation *in vivo*.

## Results

### *Jpx* transgenes induce *Xist* expression in mESCs using both *trans* and *cis* mechanisms

**Fig 1:**
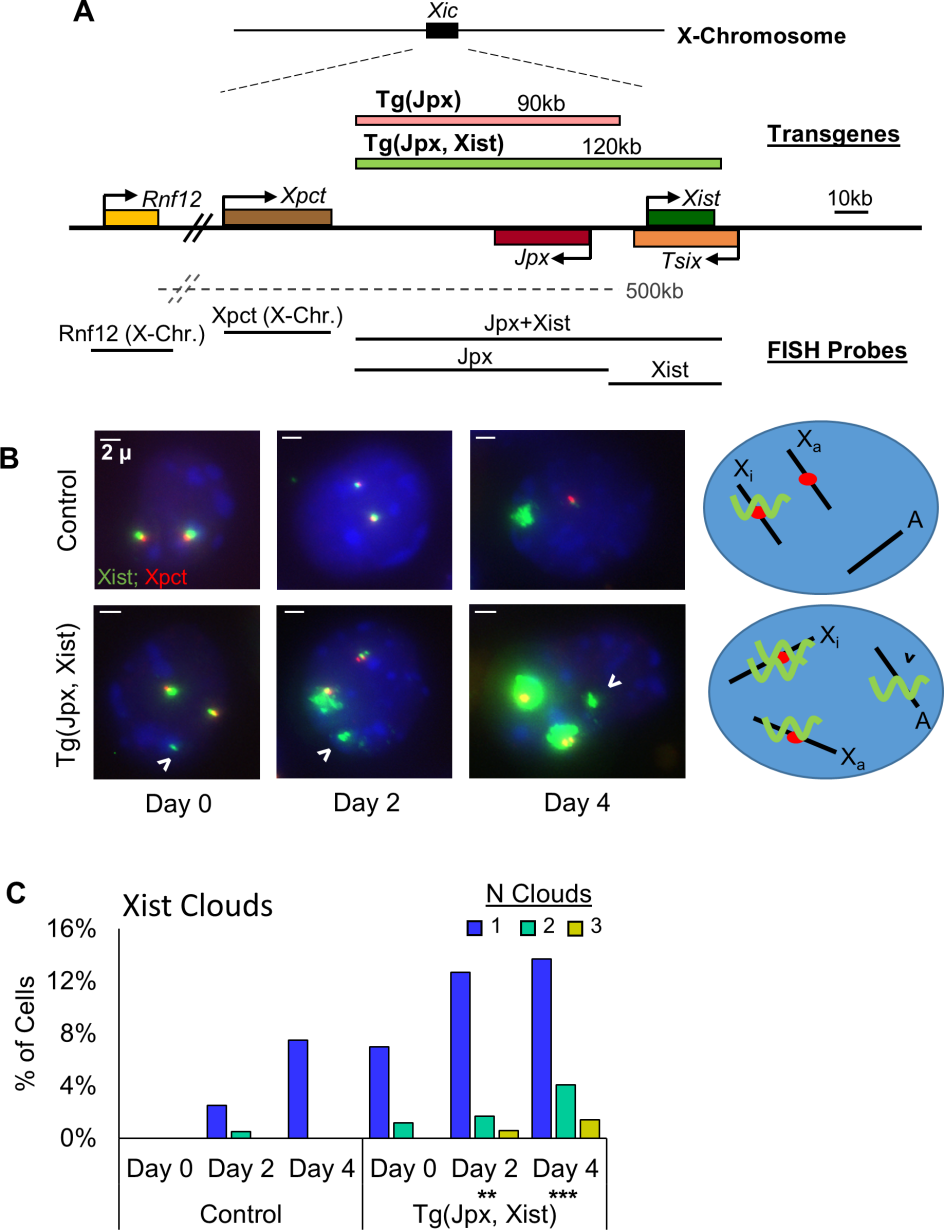
*Jpx* transgenes induce *Xist* expression in mESCs using both *trans* and *cis* mechanisms. (A) Map of the X-inactivation center (Xic) with 90kb Tg(Jpx) and 120kb Tg(Jpx, Xist) transgenes and the probes used for Fluorescence *in situ* Hybridization (FISH). (B) Combined RNA-DNA FISH for *Xist* (green, FITC) and *Xpct* (red, Cy3) on female mESC at differentiation days 0, 2, and 4. Top: Control female ESCs transfected with Tg(pSKYneo+), a plasmid that does not contain X-chromosome sequence but provides the same neomycin resistance as Tg(Jpx, Xist); bottom: Tg(Jpx, Xist) transgenic female mESC line #7, which has a single-copy Tg(Jpx, Xist) integrated in an autosome; right: schematic of *Xist* expression at Day 4. Green wavy line, *Xist* RNA; Red dot, *Xpct* (X-Chr.) DNA locus; Open arrowhead: Tg(Jpx, Xist) transgenic site. Scale bar: 2μm. (C) *Xist* cloud frequency throughout differentiation. **, *P*<0.01; ***, *P*<0.001, from a chi-square test comparing total *Xist* cloud counts in wildtype and transgenic cells at each differentiation day. Sample sizes and additional Tg(Jpx, Xist) mESC lines are included in S1 Fig.

Two transgene constructs have been generated to characterize the regulatory interaction between *Jpx* and *Xist* in mice: Tg(Jpx) is a 90kb BAC that contains the *Jpx* gene and flanking genomic DNA; Tg(Jpx, Xist) is a 120kb BAC that includes both *Jpx* and *Xist* genes in their endogenous *cis* positioning (Fig 1A). Using mESC models, a previous study inserted Tg(Jpx) into an autosome and observed ectopic *Xist* upregulation from the X chromosome in both female and male mESCs, suggesting a *trans* mechanism for *Jpx* in activating *Xist* [8]. The same Tg(Jpx), when introduced in the *Jpx*-deletion mESCs, was capable of rescuing the *Xist* and cell viability defect, which is also consistent with the proposed *trans* activity of *Jpx* [18]. In the present study, we first introduced Tg(Jpx, Xist) into mESCs to test possible mechanisms of *Xist* activation by *Jpx*. As shown in Fig 1, a single-copy Tg(Jpx, Xist) insertion in an autosome was sufficient to induce ectopic *Xist* upregulation in female ESCs. Expression of both endogenous and transgenic *Xist* were observed, suggesting both *trans*- and *cis-* effects of *Jpx* in activating *Xist*. Such effects were detected in multiple independent transgenic mESC lines, including both female and male cells carrying single-copy Tg(Jpx, Xist) insertion (S1 Fig). Stable integration of the transgene into a random autosomal site was verified by combined RNA-DNA Fluorescence *in situ* Hybridization (FISH) as in Fig 1B.

In particular, we describe the *Xist* expression pattern observed in Tg(Jpx, Xist) female mESCs upon differentiation. Combined RNA-DNA FISH was performed to visualize the characteristic *Xist* RNA “cloud” associated with the “pinpoint” DNA locus [6,18]. As shown in Fig 1B, upregulation of *Xist*, observed as an enlarged domain (green *Xist* cloud), was associated with the endogenous DNA locus (red *Xpct* pinpoint). By Day 2 of mESC differentiation, significantly more *Xist* clouds were observed in Tg(Jpx, Xist) cells compared to control cells (Figs 1C and S1A). In a small subset of cells we observed three distinct *Xist* clouds: one at each endogenous site and one at the transgenic integration site. These results support that Tg(Jpx, Xist) is functionally active in mESCs. Furthermore, ectopic *Xist* expression as two clouds at both endogenous sites demonstrate a *trans-* effect from the autosomal-integrated Tg(Jpx, Xist). In addition, an *Xist* cloud from the Tg(Jpx, Xist) transgene suggests the activation of *Xist* by its upstream *Jpx* located in *cis* within the same transgene. Such *Xist* upregulation was observed in multiple transgenic female cell lines, up to differentiation day 8 (S1B–S1D Fig), suggesting stable activation of the transgenic *Xist*. Ectopic *Xist* upregulation and transgenic *Xist* expression were also observed in male mESCs transfected with Tg(Jpx, Xist) (S1A, S1E and S1F Fig). Therefore, effects of Tg(Jpx, Xist) in mESCs indicate both *trans-* and *cis-* acting roles of *Jpx* on *Xist*.

### *Jpx* transgenes cause reduced viability of transgenic male mice

**Fig 2:**
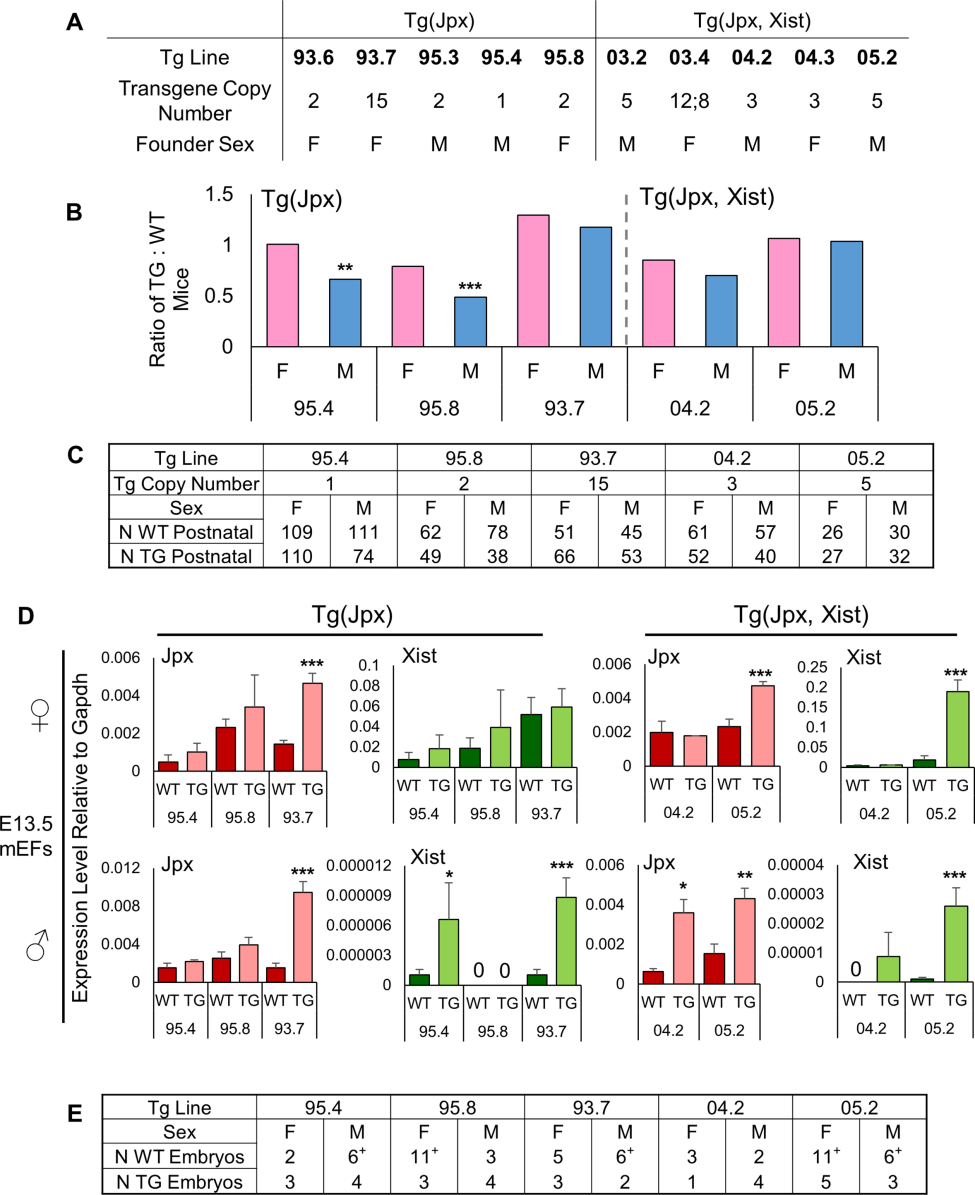
Transgene copy number is positively correlated with *Jpx* and *Xist* expression. (A) Transgenic Tg(Jpx) and Tg(Jpx, Xist) mouse lines generated in this study. F, female; M, male. In line 03.4, a semicolon separates different transgenic *Jpx* and *Xist* copy numbers. (B) The ratio of transgenic (TG) to wildtype (WT) mice for females and males obtained from five representative transgenic lines, arranged from low to high copy number for each transgene construct.**, *P*<0.01; ***, *P*<0.001, from a binomial test for the expected situation of equal TG and WT animals, independently for males and females. (C) Number of animals included in the analysis. (D) *Jpx* (red bars) and *Xist* (green bars) expression levels in mouse embryonic fibroblasts (mEFs) isolated from female embryos (top) and male embryos (bottom). Left panel: Tg(Jpx) mouse lines. Right panel: Tg(Jpx, Xist) mouse lines. Data plotted are average expression levels normalized to housekeeping gene *Gapdh*, ± standard error of biological replicates. A one-tailed *t*-test was used to compare the expression in transgenic and wildtype samples. *, *P*<0.05; **, *P*<0.01; ***, *P*<0.001. (E) Number of embryos collected and used in the expression analysis. The ‘+’ denotes the number of wildtype animals used when wildtype littermates were unavailable; expression was averaged between such wildtype animals from separate litters.

**Fig 3:**
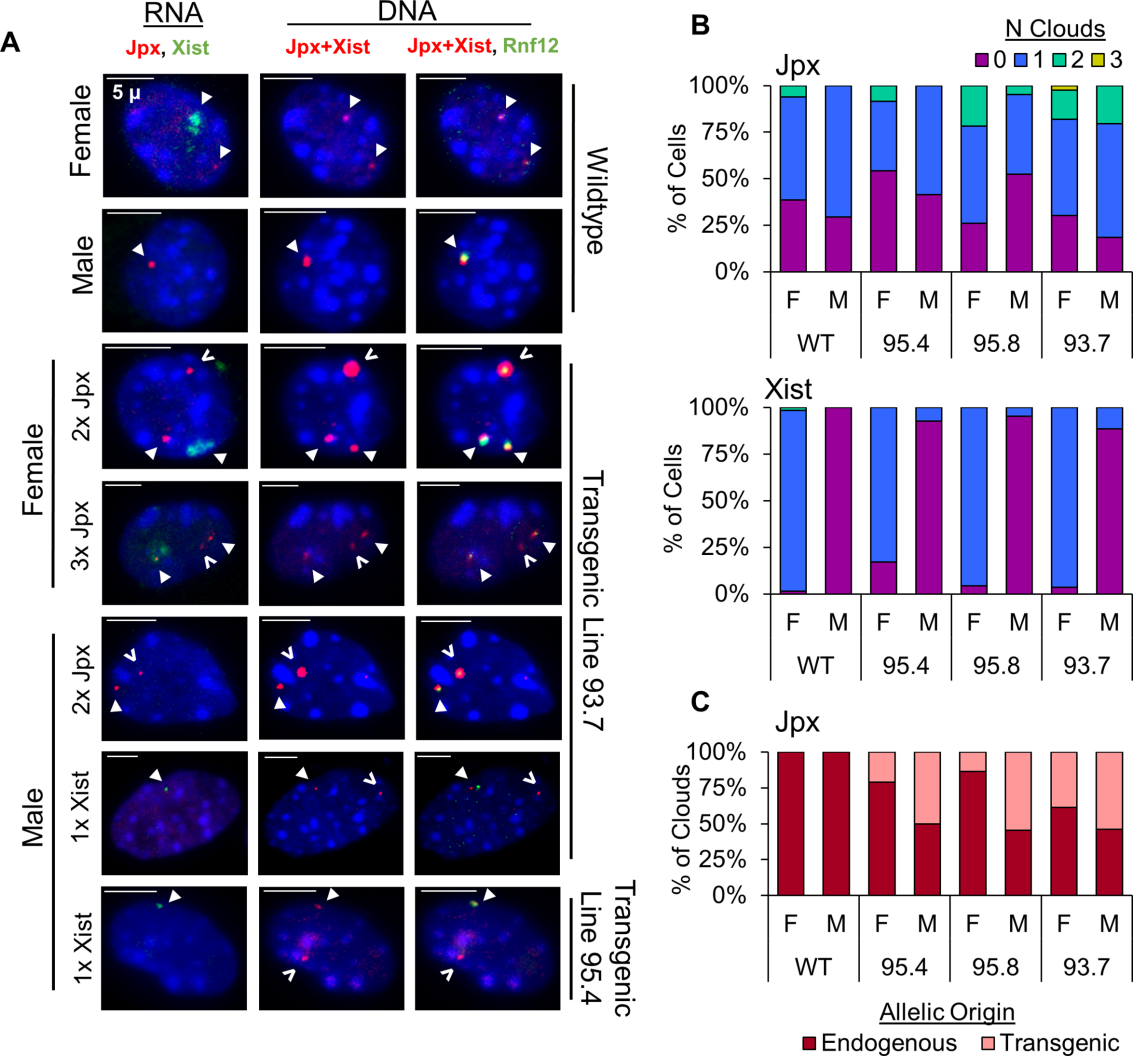
*Jpx* utilizes a *trans* mechanism to activate *Xist* expression in Tg(Jpx) mice. (A) RNA FISH (left column) and corresponding DNA FISH (two columns on the right) in wildtype and Tg(Jpx) transgenic mEFs. Representative images shown of ectopic expression patterns observed in cells. Probes are described in Fig 1A. For RNA: *Jpx* (red, Cy3) and *Xist* (green, FITC). For DNA: *Jpx*+*Xist* (red, Cy3) and *Rnf12* (green, FITC). Right column: DNA FISH with two probes to distinguish the endogenous X chromosomal locus (overlapping red and green) from the transgenic insertion site (red only). Closed arrowhead: endogenous RNA transcripts (RNA FISH) and the endogenous X chromosomal loci (DNA FISH). Open arrowhead: transgenic RNA transcripts and the transgenic integration site. Scale bar: 5μm. (B) Percentage of cells with *Jpx* or *Xist* expression categorized by the number of RNA clouds detected. (C) Percentage of endogenous versus transgenic *Jpx* clouds counted in Tg(Jpx) mEFs.

**Fig 4:**
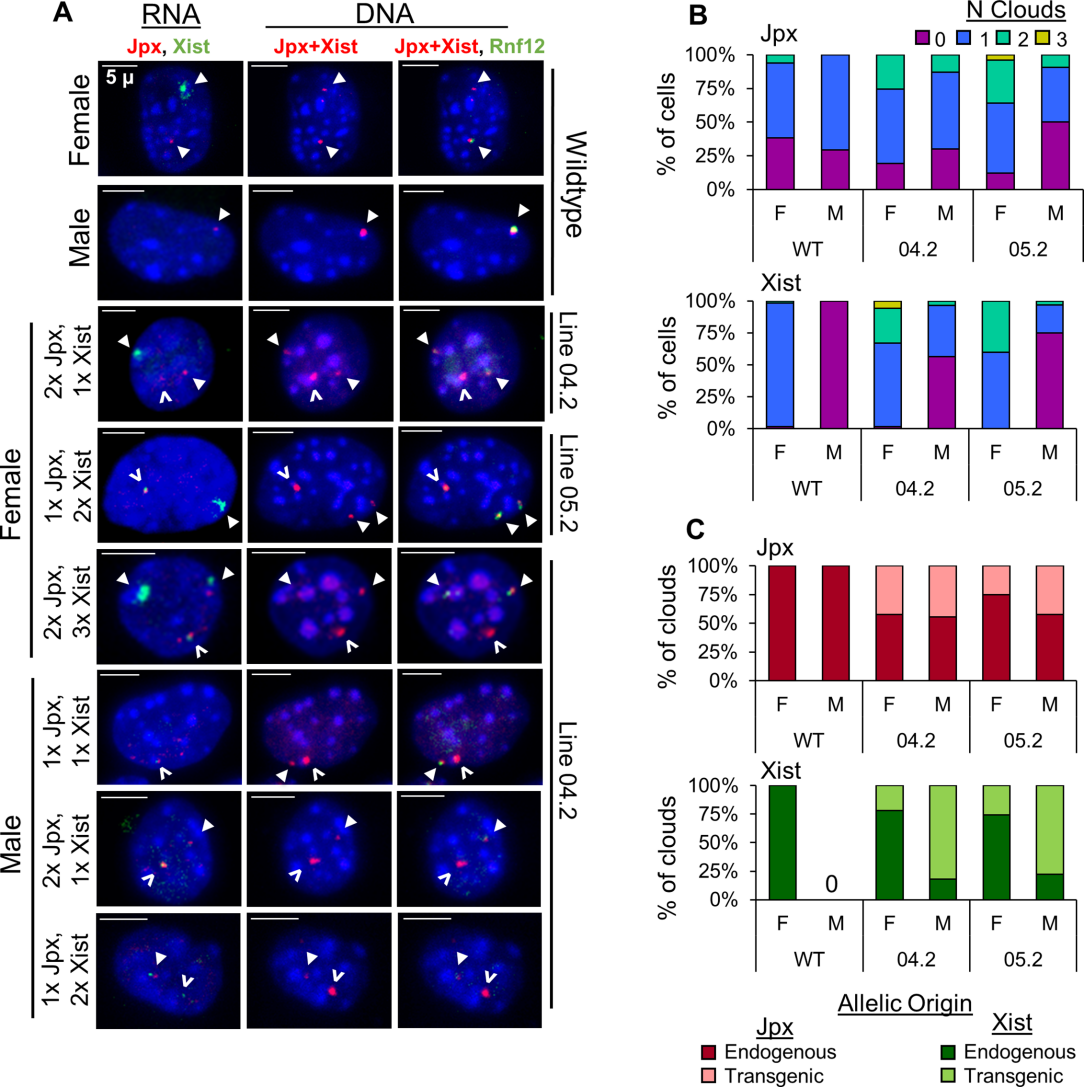
*Jpx* activates *Xist* expression using both *cis* and *trans* mechanisms in Tg(Jpx, Xist) mice. (A) RNA FISH (left column) and corresponding DNA FISH (two columns on the right) in transgenic Tg(Jpx, Xist) mEFs. Representative images shown of ectopic expression patterns observed in cells. Probes are described in Fig 1A. For RNA: *Jpx* (red, Cy3) and *Xist* (green, FITC); for DNA: *Jpx*+*Xist* (red, Cy3) and *Rnf12* (green, FITC). Right column: DNA FISH with two probes to distinguish the endogenous X chromosomal locus (overlapping red and green) from the transgenic insertion site (red only). Closed arrowhead: endogenous RNA transcripts (RNA FISH) and the endogenous X chromosomal loci (DNA FISH). Open arrowhead: transgenic RNA transcripts and the transgenic integration site. Scale bar: 5μm. (B) Percentage of cells with *Jpx* or *Xist* expression categorized by number of RNA clouds detected. (C) Percentage of endogenous versus transgenic RNA clouds for *Jpx* and *Xist* in Tg(Jpx, Xist) mEFs.

By introducing Tg(Jpx) or Tg(Jpx, Xist) as transgenes in mice, we next asked if *Jpx* would be sufficient to induce *Xist* expression and whether increasing *Jpx* gene copy number leads to any observable abnormality in live animals. Transgenic mice were generated by microinjecting BAC DNA into mouse pronuclei and were recovered as founder animals for independent lines. Fig 2A summarizes a total of ten transgenic mouse lines obtained, five for Tg(Jpx) and five for Tg(Jpx, Xist), with copies of transgenes randomly integrated in the genome. All transgenic founder mice appeared morphologically normal, fertile, and were able to transmit the transgene to the next generation. Stable integration and inheritance of the transgenes were verified by genotyping offspring along five generations of outcrossing. For each transgenic line, a single autosomal integration site was detected by DNA FISH localizing the transgene in mouse fibroblasts, as exampled in Figs 3 and 4. Five representative lines, with transgene copy numbers ranging from one to fifteen, were characterized to address the regulatory effects of *Jpx* on *Xist* in this study.

Transgenic animals of both sexes were born in each line, yet with variable frequencies as compared to wildtype littermates. In Fig 2B, we plotted the ratios of transgenic to wildtype (TG: WT) animals born within each line and found an overall difference between the male (TG/WT ratio average = 0.81) and female (TG/WT ratio average = 1.00). A paired student *t-*test indicated a significantly lower representation of transgenic males (the one-tailed *P* = 0.01). The male viability defect was most obvious in two independent Tg(Jpx) lines, 95.4 and 95.8, in which nearly 50% fewer transgenic males were born (Fig 2B and 2C). A male-specific viability defect suggests possible influence to the X chromosome: transgenic *Jpx* may be activating the single endogenous *Xist* on the only male X chromosome. Ectopic activation of *Xist* may lead to inappropriate silencing of X-linked essential genes in the male. By contrast, the same effect to endogenous *Xist* in the female could be modulated between its two X’s, thus minimizing the potentially deleterious consequence of silencing both X chromosomes.

### Transgene copy number is positively correlated with *Jpx* and *Xist* expression

We compared *Jpx* and *Xist* expression levels in five transgenic lines with different copy numbers of the transgene. To specifically focus on gene activities in embryonic tissues, we isolated mouse embryonic fibroblasts (mEFs) from embryonic day 13.5 (E13.5). As shown in Fig 2D, both *Jpx* and *Xist* transcript levels increased with increasing transgene copy number, reflecting a dose-dependent gene regulation. At the same time, higher *Jpx* levels correlated with increased *Xist* expression, suggesting positive regulation of *Jpx* on *Xist* in these transgenic animals. In particular, *Xist* expression was detected in male mEFs of transgenic lines 95.4, 93.7, 04.2 and 05.2, whereas wildtype males normally have no *Xist* expression in any somatic cell (Fig 2D). A control qRT-PCR reaction performed without reverse-transcriptase (RT minus) confirmed that *Xist* amplification detected in the Tg(Jpx) male was indeed of *Xist* RNA (S2A Fig). We also noted variability in *Jpx* and *Xist* expression between littermate animals of the same genotype, and between litters of the same lineage (Fig 2D, standard errors). Thus, littermates of wildtype and transgenic animals were used for all comparisons, except for noted situations (Fig 2E; denoted by ‘+’) when wildtype littermates were not available and an average of wildtype samples was used in analysis.

In Tg(Jpx, Xist) transgenic animals, increased *Xist* expression was observed from both females and males in line 05.2. While this ectopic *Xist* likely derives from the transgene, the transcripts cannot be distinguished from endogenous *Xist* by nucleotide sequence or the qRT-PCR assay in this analysis. In contrast, Tg(Jpx) does not contain *Xist*, and thus the observed induction of *Xist* expression in 95.4 and 93.7 transgenic males must be exclusively *trans*-activation of the endogenous *Xist* (the only copy on the male X chromosome) by the transgenic *Jpx* (from the autosomal Tg(Jpx)). Therefore, these data demonstrate that increasing *Jpx* gene copies induces *Xist* expression in mice, and that transgenic *Jpx* can activate the endogenous *Xist* in *trans*.

### *Jpx* utilizes a *trans* mechanism to activate *Xist* expression in Tg(Jpx) mice

To clearly distinguish between transgenic and endogenous gene activation, we performed sequential RNA and DNA FISH on Tg(Jpx) E13.5 mEFs. This technique allowed independent visualization of *Jpx* and *Xist* transcripts and of the genomic transgene integration site (Fig 3A). Sequential FISH on the same cell allowed us to associate an RNA cloud with its corresponding DNA locus on the endogenous or transgenic allele (Fig 3A and 3B: closed arrowhead, endogenous; open arrowhead, transgenic). On its own, DNA FISH was also used to confirm the correct ploidy and X chromosome number in each cell. In high transgene copy lines (such as line 93.7), the transgenic DNA locus often appeared much larger and was distinct from the smaller endogenous loci, as visualized by the ‘*Jpx*+*Xist*’ probe (Fig 3A, middle column). To confirm endogenous alleles when the transgene was not obvious, we used a second probe, ‘*Rnf12*,’ to target the X chromosome outside the transgene sequence (Fig 1A), which co-localized with endogenous *Jpx* and *Xist* but not with the transgenic allele (Fig 3A, right column). Taken together, sequential RNA and DNA FISH allowed us to identify the expression pattern of *Jpx* and *Xist* in Tg(Jpx) mEFs.

Wildtype mEFs typically displayed *Jpx* RNA FISH foci larger than a single pinpoint, which we denote as a dot-like ‘cloud’ to signify the expression of *Jpx* from the gene locus. As shown in Fig 3, wildtype female mEFs displayed one *Jpx* RNA cloud and one *Xist* RNA cloud, each corresponding to an endogenous gene locus; wildtype male mEFs displayed one *Jpx* RNA cloud at the endogenous *Jpx* gene locus and showed no *Xist* RNA. In contrast, additional *Jpx* RNA clouds were observed in female and male Tg(Jpx) transgenic mEFs (Fig 3A and 3B), consistent with our finding of increased *Jpx* expression when *Jpx* gene copies are increased (Fig 2). Since the total number of cells expressing *Jpx* remained comparable between wildtype and transgenic (S2B Fig), the data suggest that the increase in *Jpx* expression measured by qRT-PCR was due to increased *Jpx* expression at the individual cell level. We then quantified the *Jpx* cloud allelic origin in Fig 3C and found robust *Jpx* expression from the transgene—on average, 25% of female *Jpx* clouds and 50% of male *Jpx* clouds were transgenic—reaffirming that the BAC transgene contains all regulatory elements sufficient for *Jpx* expression in mice, and that transgenic expression is likely responsible for the observed increase in *Jpx* transcript levels.

*Xist* expression in Tg(Jpx) E13.5 mEFs was also affected by the increase in *Jpx* copy number (Fig 3A and 3B). FISH on transgenic female mEFs revealed only one *Xist* RNA cloud—originating from one of the two endogenous X chromosomes—even in the presence of supernumerary *Jpx* expression (Fig 3A, middle panels). This indicates that Tg(Jpx) E13.5 transgenic females maintain proper dosage compensation with only one silenced X chromosome, which is in agreement with our finding of normal viability in these females (Fig 2B). The increase in *Xist* expression detected by qRT-PCR (Fig 2D) suggests enhanced *Xist* transcription from X_i_, likely affected by *Jpx* acting in *trans* from the transgenic site. In comparison, an ectopic *Xist* cloud was observed on the only X chromosome in Tg(Jpx) male cells (Fig 3A, lower panels; Figs 3B and S2B). This observation is consistent with the detected *Xist* transcripts and viability reduction observed in such transgenic males (Fig 2B and 2D). Overall, by combining RNA and DNA FISH results in the same cells, we confirmed the autosomal integration of transgenes and the allelic association of *Jpx* and *Xist* transcripts. Transgenic *Jpx* induced endogenous *Xist* expression in both male and female mEFs, thus demonstrating a *trans* mechanism of activation in mice.

### *Jpx* activates *Xist* expression using both *cis* and *trans* mechanisms in Tg(Jpx, Xist) mice

The Tg(Jpx, Xist) transgene includes *Jpx* and *Xist* genomic sequences in their endogenous *cis* positioning, as illustrated in Fig 1A. DNA FISH in Tg(Jpx, Xist) mEFs confirmed the single-site autosomal integration of transgenes, and sequential RNA and DNA FISH resolved the allelic origin of *Jpx* and *Xist* transcripts in individual cells (Fig 4A). As the only two genes contained in Tg(Jpx, Xist), *Jpx* using *cis* mechanisms to regulate *Xist* expression would lead to expression of both genes from the same transgenic allelic locus. Indeed, we observed activation and co-localization of *Jpx* and *Xist* RNA associated with the transgenic site in both female and male Tg(Jpx, Xist) mEFs (Fig 4A: open arrowheads). Significantly more transgenic female cells expressed *Jpx* compared to wildtype controls (Figs 4B and S2B). This increase in *Jpx* activity is likely due to expression from the transgenic locus, as approximately 30% of all *Jpx* clouds in Tg(Jpx, Xist) females were transgenic in origin (Fig 4C). Consequently, transgenic *Xist* clouds represented close to 30% of all observed *Xist* clouds in Tg(Jpx, Xist) females (Fig 4C). The percentage of detectable *Jpx* clouds in transgenic male cells was comparable to wildtype, and males maintained a 50/50 split between endogenous and transgenic *Jpx* activation (Figs 4C and S2B). Importantly, more than 75% of *Xist* clouds in males were transgenic, contributing to a significantly higher percentage of *Xist* clouds detected in transgenic male cells compared to wildtype controls (Figs 4C and S2B). These data are consistent with our observation of increased *Jpx* and *Xist* expression in Tg(Jpx, Xist) males (Fig 2D).

Endogenous *Xist* was also affected in Tg(Jpx, Xist) transgenic mEFs: we observed three *Xist* clouds in a female cell and two *Xist* clouds in a male cell, representing ectopic *Xist* activation on the X chromosome (Fig 4A: arrowheads, Fig 4B). Within one cell, ectopic *Xist* activation from the endogenous X chromosome is consistent with a *trans* regulatory response from the autosomal transgene, while the concurrent *Xist* and *Jpx* expression from the transgenic allele suggests a *cis* activation of *Xist* by the flanking *Jpx*. We also note that the endogenous *Jpx* may induce transgenic *Xist* expression through a *trans* mechanism in the same cell. When we examined the allelic origin of ectopic *Xist*, as a single *Xist* cloud in the male or a second cloud in the female, we found that an *Xist* cloud was more often associated with the transgene than with the endogenous X chromosome (S2C and S2D Fig), suggesting that transgenic *Xist* activation contributes to the increase of *Xist* expression observed in Tg(Jpx, Xist) mice (Fig 2D). Tg(Jpx, Xist) has thus demonstrated *Jpx* activation of *Xist* expression *in vivo*, and revealed the possible mechanism as cooperating both *cis* and *trans* activities.

### Ectopic *Xist* silences X-linked genes in Tg(Jpx) mice

**Fig 5:**
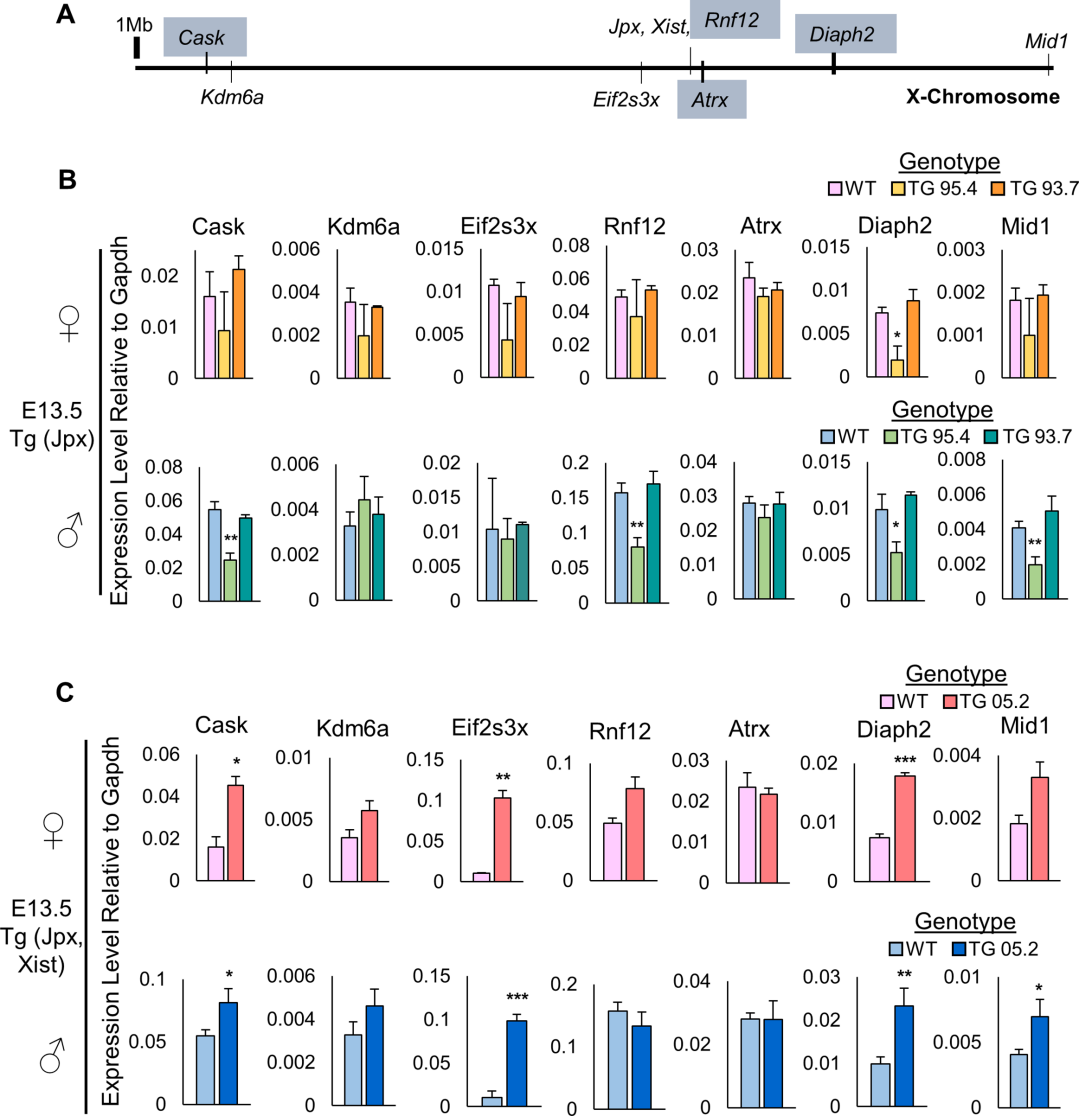
Ectopic *Xist* silences X-linked genes in Tg(Jpx) transgenic mice. (A) Map of X chromosome and genes for quantitative expression analysis in E13.5 mEFs. Genes boxed in grey (*Cask*, *Rnf12*, *Atrx*, *Diaph2*) are subject to XCI in mice; genes not boxed (*Kdm6a*, *Eif2s3x*, *Jpx*, *Xist*, *Mid1*) are known to escape XCI in mice. (B) Expression of X-linked genes in wildtype and Tg(Jpx) transgenic mEFs isolated from lines 95.4 and 93.7. Top: Expression in females. Bottom: Expression in males. (C) Expression of X-linked genes in wildtype and Tg(Jpx, Xist) transgenic mEFs isolated from line 05.2. Top: Expression in females. Bottom: Expression in males. Data plotted are average expression levels normalized to housekeeping gene *Gapdh*, ± standard error of biological replicates. A subset of embryos from Fig 2E were used for analysis, N≥2 for each genotype. A one-tailed *t*-test was used to compare the expression in transgenic and wildtype samples. *, *P*<0.05; **, *P*<0.01, ***, *P*<0.001.

We next asked if the observed ectopic *Xist* expression would induce additional XCI and silence X-linked genes in our transgenic animals. We performed quantitative expression analysis (qRT-PCR) for seven X-linked genes, which are located across the length of the X-chromosome at positions of various distance from *Xist* (Fig 5A). Of these seven genes, *Cask*, *Rnf12*, *Atrx*, and *Diaph2* are genes subject to XCI in females (Fig 5A, boxed grey) while *Kdm6a*, *Eif2s3x*, and *Mid1* are genes known to escape XCI in mice [20,21]. As shown in Fig 5B, we observed an overall reduction of X-linked gene expression in Tg(Jpx) E13.5 mEFs from the line 95.4 compared to wildtype mEFs. A significant reduction of *Diaph2* expression was observed in Tg(Jpx) line 95.4 for both female and male mEFs. Particularly in the males, four out of the seven X-linked genes, including *Cask*, *Rnf12*, *Diaph2* and *Mid1*, were significantly downregulated in line 95.4. Such a decrease of X-linked gene expression indicates a gene silencing effect, most likely from the ectopic *Xist* expression, in the Tg(Jpx) 95.4 transgenic animals. An overall downregulation of X-linked genes may lead to developmental disadvantages, which is consistent with the lack of transgenic males observed in line 95.4 (Fig 2B). By contrast, there was no reduction of X-linked gene expression in the mEFs of Tg(Jpx) line 93.7, consistent with a normal survival rate of transgenic animals in this line (Fig 2B). These data demonstrate that ectopic *Xist* expression induced by Tg(Jpx) indeed has functional consequence in silencing X-linked genes, which may lead to physiological defects affecting male viability in mice.

Tg(Jpx, Xist) mEFs did not display the same X-linked gene silencing effect. Instead, an overall increase of X-linked genes was observed in both male and female E13.5 cells from Tg(Jpx, Xist) line 05.2 (Fig 5C). We noted that ectopic *Xist* expression in Tg(Jpx, Xist) mEFs was observed with higher frequency on the autosomal transgene than the endogenous X chromosome (S2C and S2D Fig). Therefore, *Xist* upregulation in Tg(Jpx, Xist) cells may preferably affect autosomal genes flanking the transgene integration site rather than silencing the endogenous X chromosome. Transgenic *Xist* in an autosome has been shown to be capable of silencing autosomal genes in *cis* [22]. At the same time, robust transgenic *Xist* expression can also squelch the endogenous *Xist* [23], potentially affecting the X-linked gene silencing. Upregulation of X-linked genes with XCI deficiency is compatible with mouse survival [24]. This is also consistent with the observed viability of Tg(Jpx, Xist) animals (Fig 2B).

### Ectopic *Xist* expression in transgenic female and male early embryos

**Fig 6:**
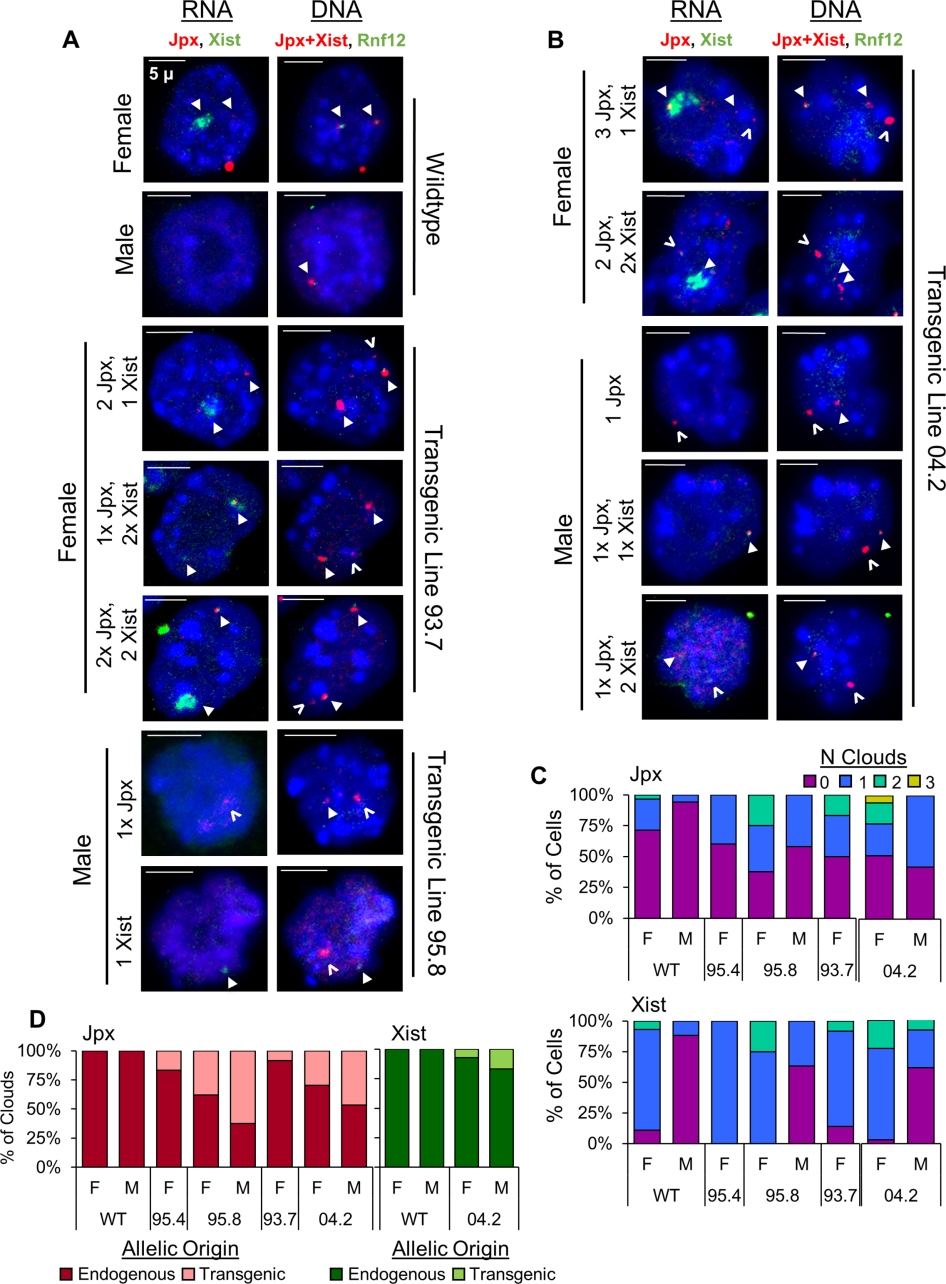
Ectopic *Xist* expression in transgenic female and male early embryos. (A, B) RNA FISH (left column) and corresponding DNA FISH (right column) in wildtype, transgenic Tg(Jpx) (A), and transgenic Tg(Jpx, Xist) embryos (B), extracted at embryonic day 7.5 (E7.5). Representative images shown of ectopic expression patterns observed in cells. Probes are described in Fig 1A. For RNA: *Jpx* (red, Cy3) and *Xist* (green, FITC); for DNA: *Jpx*+*Xist* (red, Cy3) and *Rnf12* (green, FITC). DNA FISH with two probes distinguishes the endogenous X chromosomal locus (overlapping red and green) from the transgenic insertion site (red only). Closed arrowhead: endogenous RNA transcripts (RNA FISH) and the endogenous X chromosomal loci (DNA FISH). Open arrowhead: transgenic RNA transcripts and the transgenic integration site. Scale bar: 5μm. (C) Percentage of cells with *Jpx* or *Xist* expression categorized by number of RNA clouds detected. (D) Percentage of endogenous versus transgenic RNA clouds for *Jpx* and *Xist* in Tg(Jpx) and Tg(Jpx, Xist) embryos. Number of E7.5 embryos and quantification are included in S3A Fig.

XCI occurs in the mouse embryo between embryonic days 5.5 (E5.5) and 6.5 (E6.5) [25]. We asked whether the effects of transgenic *Jpx* on *Xist* could be more apparent in the early embryos around the completion of XCI. To address this question, we extracted post-implantation E7.5 and E8.5 embryos, wildtype and transgenic littermates, from Tg(Jpx) lines, 93.7 and 95.8, and Tg(Jpx, Xist) line 04.2 (Figs 6 and S3). Specifically, we analyzed cells isolated from the embryo proper, where random XCI occurs [26]. As shown in Fig 6, compared to the wildtype male and female embryonic cells, transgenic male and female E7.5 embryonic cells showed ectopic *Xist* expression. Notably in Tg(Jpx) transgenic female cells, we observed two *Xist* clouds present in up to 25% of cells (Fig 6A and 6C). Occurrence of two *Xist* clouds in a cell was never observed in any Tg(Jpx) female E13.5 mEF (Fig 3B). These data further support a *trans-* effect of *Jpx* (from the autosomal transgene) in activating both *Xist* alleles on the X chromosomes, which may lead to cell death during early embryogenesis. Ectopic expression of endogenous *Xist* was also observed in Tg(Jpx) transgenic male E7.5 cells, consistent with the pattern in E13.5 mEFs, which confirms the *trans-* acting effect of *Jpx* on *Xist* activation in these embryos.

Also consistent with the expression pattern of *Jpx* and *Xist* in Tg(Jpx, Xist) E13.5 mEFs, Tg(Jpx, Xist) female and male E7.5 embryonic cells showed expression of both endogenous and transgenic *Jpx* clouds, which were associated with up to two *Xist* clouds, one endogenous and one transgenic (Fig 6B–6D; S3A Fig). We note that even with limited cell samples obtained from E7.5 embryos, ectopic *Xist* RNA was clearly present in the early embryonic cells from transgenic mice–confirming our observation of ectopic *Xist* expression in E13.5 transgenic mEFs. Together, these data suggest a mechanism in which *Jpx* is sufficient to induce *Xist* expression in *trans* (from autosomal transgenic *Jpx* to the endogenous *Xist* on X chromosomes) and is capable of activating *Xist* in *cis* (locally within a transgene).

### Discussion

**Fig 7:**
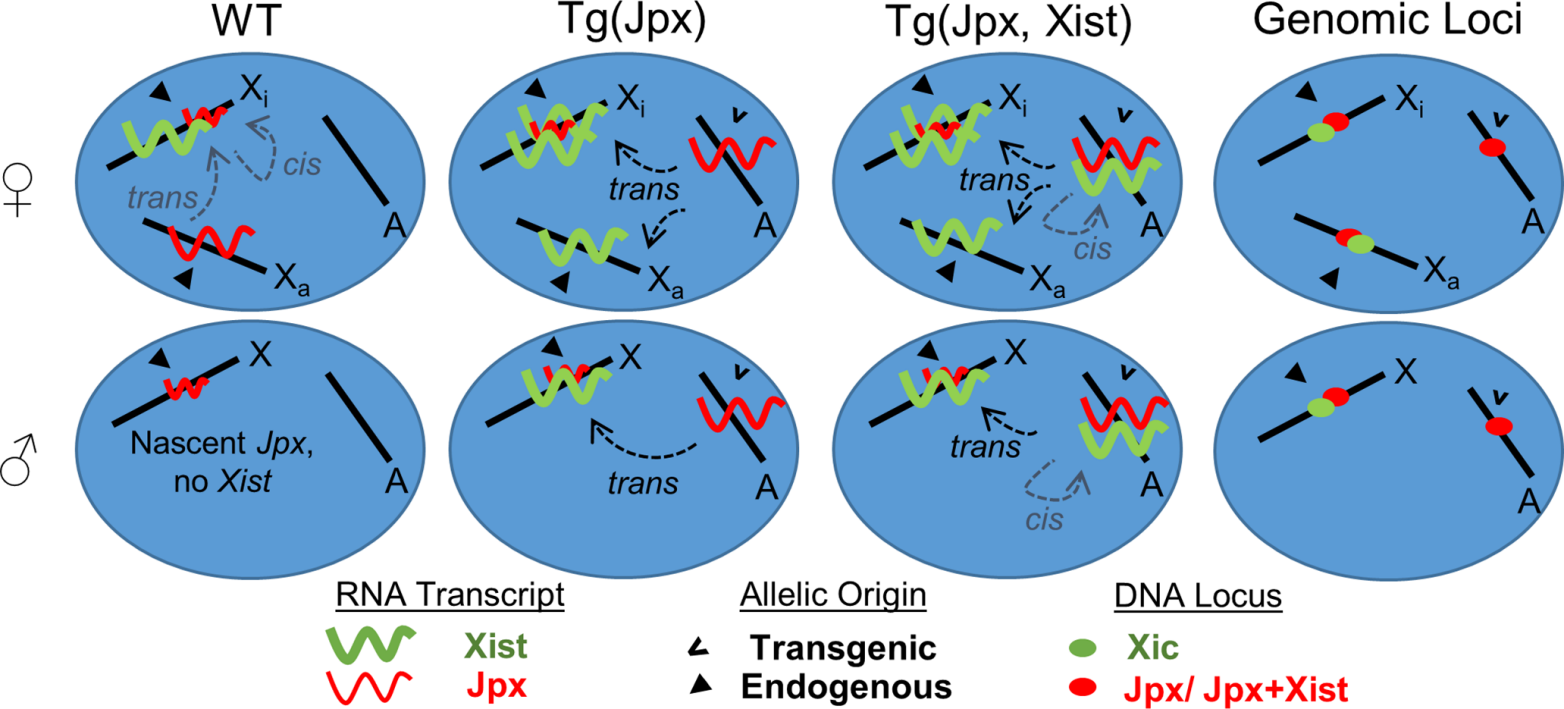
*Jpx* activates *Xist* expression in transgenic mice. Summary and model for how *Jpx* activates *Xist* in wildtype (WT) and transgenic mice. The grey dashed arrows in WT represent the proposed mechanisms for *Jpx* activating *Xist*. Up to two endogenous *Xist* clouds were observed in Tg(Jpx) embryos, indicating *trans* activity by *Jpx* (black dashed arrows). In Tg(Jpx, Xist) embryos, up to three *Xist* clouds were observed: two endogenous and one transgenic. This suggests *Jpx* regulation of *Xist* using the proposed *cis* mechanism (grey dashed arrows) in addition to the *trans* mechanisms (black dashed arrows).

Our findings suggest a model in which *Jpx* is a competence factor that initiates *Xist* expression in mice (Fig 7). By using a combination of transgenes, we demonstrated that increasing *Jpx* copy number is sufficient to activate *Xist* expression in mice. The *Jpx* transcript is a *trans*-acting factor which, when increased by addition of the Tg(Jpx) transgene in an autosome, is capable of activating endogenous *Xist* on the X chromosome in both male and female mice. In addition, *Jpx* has been described as a member of the *Xist* topologically associated domain (TAD) [27,28], indicating *cis* regulatory activity for *Jpx* inducing *Xist* locally within the same chromosomal locus [29]. This is consistent with our observation of co-localized *Jpx* and *Xist* transcripts from the Tg(Jpx, Xist) transgene in mice, suggesting that *Jpx* activates *Xist* expression in *cis* within the transgene. Together, our observations in Tg(Jpx) and Tg(Jpx, Xist) mice illustrate *Jpx* inducing *Xist* expression, and support *Jpx* as a competence factor directly influencing X chromosome counting and XCI initiation.

To distinguish between *trans*- and *cis-* genetic mechanisms in our mouse models, we emphasize the distinction between *inter-* and *intra-* chromosomal gene regulation. Therefore, an autosomal *Jpx* transgene activating the endogenous *Xist* on the X chromosome is a clear demonstration of *trans-* acting function of *Jpx*; whereas the activation of a transgenic *Xist* by its upstream *Jpx* within the same transgene locus is considered a *cis*- effect of *Jpx*. Our results suggest that *Jpx* can activate *Xist* locally within the transgene; however, it is possible that *Jpx* RNA moves away from its site of transcription and returns to the target *Xist* locus. At the molecular scale, this mechanism would be considered *trans* acting; whereas the genetic effect is considered *cis* regulation. At the *Xist* promoter, the chromatin insulating factor CTCF has been shown to bind *Jpx* RNA, and together, act in a dose-dependent mechanism for transcriptional initiation of *Xist* in female mESCs [8]. Titration of CTCF from the *Xist* promoter requires both copies of the *Jpx* gene and *Jpx* RNA transcribed from both X chromosomes in a female cell, consistent with a combination of both *cis* and *trans* mechanisms for *Jpx* RNA. For chromosomal configuration around the *Xist* locus, a change in CTCF occupancy could alter TAD boundary formation, which may facilitate a *cis* interaction between the *Xist* gene and flanking *Jpx*. As recently reported, other proteins in addition to CTCF may also play roles for the formation of TADs in the X chromosome [30]. Additionally, unknown protein factors may be actively directing the *trans* activity of *Jpx* RNA between chromosomes. Future research in identifying *Jpx-*protein binding partners will help elucidate the possible molecular interactions, which are most likely developmentally regulated in mice.

While we observed an overall positive correlation between *Jpx* copy number and expression levels, we did not observe an obvious increase of *Jpx* RNA FISH signals associated with high-copy *Jpx* transgenes, i.e. Tg(Jpx) 93.7 (Fig 3A). A feedback mechanism may regulate the allelic expression of high-copy *Jpx* to inhibit unfavorable allelic overexpression in a cell. This possibility is also supported by the observed *Jpx* allelic expression in transgenic mESCs, in which addition of ‘*Jpx/Ftx* transgene’ actually decreased the endogenous *Jpx* expression (Figure S4A in Barakat et al., 2014) [10]. In addition, the activation of *Xist* within the Tg(Jpx, Xist) transgene could also be complemented by the lack of a functional *Tsix*, a suppressor of *Xist* [6,18], which might facilitate a preference of transgenic *Xist* expression over the endogenous *Xist* in the X chromosomes. It is also possible that the endogenous *Jpx trans*- activates the transgenic *Xist*, or works together with the transgenic *Jpx* to cooperate both *trans* and *cis* mechanisms when activating transgenic *Xist* expression in Tg(Jpx, Xist) animals.

High percentages of transgenic *Xist* expression were observed in Tg(Jpx, Xist) E13.5 mEFs (Fig 4C). However, in earlier embryonic cells from E7.5, the majority of *Xist* clouds observed were endogenous in origin (Fig 6D). To understand whether upregulation of the endogenous *Xist* induced XCI in early embryos, we performed qRT-PCR on seven X-linked genes in Tg(Jpx) lines 95.8 and 93.7. As shown in S3B–S3E Fig, there was no obvious decrease of X-linked gene expression in Tg(Jpx) E8.5 or E7.5 embryos. This is in contrast to the significant reduction of X-linked genes observed in Tg(Jpx) E13.5 mEFs (Fig 5). It is possible that the additional silencing effect by ectopic *Xist* was achieved and only obvious at a later stage of the typical XCI developmental timing in mouse embryogenesis.

Overall, our transgenic system provides an example linking *ex vivo* and *in vivo* studies of lncRNA function and mechanism. While gene knockout would be a conventional approach for functional determination, recent reports on the function of lncRNA *Hotair* revealed the complications of lncRNA deletion in mice [31–34]. The debate on *Hotair*’s molecular mechanism and target genes also advocates for the use of transgenic mice when distinguishing *cis-* and *trans-* actions of lncRNA [32,33]. In this study, our transgenic mouse models help resolve *Jpx*’s function and mechanism while avoiding possible genomic instability brought about by large deletions of lncRNA genes. Our study thus provides an example of lncRNA functional studies in mice and demonstrates that *Jpx* is sufficient to activate *Xist* expression using *trans* and *cis* mechanisms *in vivo*.

## Materials and methods

### Ethics statement

Mice were housed at the University of California, Irvine and handled according to Institutional Animal Care and Use Committee (IACUC) guidelines. Animal Use Protocol number: 2013–3109.

### Transgenic embryonic stem cells

The Tg(Jpx, Xist) transgene was subcloned from BAC 388K20, a BAC8 transgene [35,36] via ET-Cloning. The transgene was introduced into male (J1) and female (16.7) ES cells by electroporation, and positive clones were picked under neomycin antibiotic (G418, Geneticin, Gibco, Life Technology) selection. DNA FISH was used to confirm stable transgene integration. Control cells were obtained from a parallel electroporation procedure using a pSKYneo+ plasmid, which does not contain any X chromosome sequence but provides the same neomycin resistance to selected clones. Mouse ES cells were differentiated as described previously [8].

### Generation of transgenic mice

The Tg(Jpx) and Tg(Jpx, Xist) transgene constructs were subcloned from a BAC8 transgene [35,36]. DNA was purified using a Macherey-Nagel NucleoBond Xtra BAC kit. Pronuclear injection of DNA into B6SJLF1/J donor embryos was performed at the UCI Transgenic Mouse Facility. Transgenic founder animals were crossed with C57BL/6J wildtype mice to establish individual transgenic lines. Crosses performed were WT/WT x TG/WT (TG: Transgenic; founders of both sexes were obtained and used as transgenic donors). Mice were identified as transgenic by genomic PCR on toe DNA using a primer set against the BAC8 vector sequence [36]. Mouse gender was determined by priming to the UBEX gene [37].

### Quantitative PCR

To determine transgene copy number, genomic DNA was isolated and purified from lysed toe tissue and primed for genomic *Jpx* and *Xist* genes. Presence of the transgene was also confirmed by priming to the BAC8 vector [36]. Transgene copy number was determined by normalizing the genomic *Jpx* and *Xist* copy numbers to the X-linked *Hprt* gene (internal control) and comparing to the wildtype male or female samples. To measure RNA expression, cultured E13.5 mEFs or minced embryo tissue (E7.5/ 8.5) were homogenized in TRIzol Reagent (Invitrogen); chloroform and isopropanol were used to extract and precipitate RNA; and the RNA was treated with TURBO DNaseI (Life Technology) before reverse transcription with Maxima Reverse Transcriptase (Thermo Fisher). qRT-PCR was then performed on a BioRad CFX96 Real-Time PCR system. *Jpx* and *Xist* primers targeted mature transcripts as described in the Supplemental Experimental Procedures. *Gapdh* expression was used as an internal control [38].

### Mouse embryonic fibroblast (mEF) extraction

Mouse mating was timed such that embryos were extracted on embryonic day 13.5 (E13.5). Briefly, each pup’s head was removed and used for genotyping. The organs (lungs, heart, liver, GI tract) were removed and the body cavity (containing fibroblasts) was diced and stored overnight in 0.25% Trypsin-EDTA (Gibco, Life Technologies) at 4°C. Tissue chunks were digested for 20 minutes at 37°C and filtered through a 70μm nylon Falcon cell strainer (Fisher). Cells were passaged once to select for live mEFs. Half of the culture was then resuspended in TRIzol Reagent for RNA extraction and qRT-PCR while the other half was cryopreserved or cultured further for FISH.

### Post-implantation embryo extraction

Mouse mating was timed such that embryos were extracted on embryonic day 7.5 (E7.5) or day 8.5 (E8.5) as described in [39]. Briefly, whole embryos were isolated from the pregnant mother’s uterus and separated from the decidua. Using a scalpel, the embryo was cut to separate the embryo proper from the extra-embryonic tissues. For embryos used in FISH, the embryo proper was minced well with the scalpel and soaked in 0.25% Trypsin-EDTA (Gibco, Life Technologies) for approximately 1 hour at 4°C. The embryonic tissues were then digested for 10 minutes at 37°C and homogenized via pipetting, then cytospun onto two slides per embryo and fixed in 4% paraformaldehyde. For embryos used in qRT-PCR, the embryo proper was homogenized directly in TRIzol Reagent (Invitrogen). RNA was extracted as described above. E8.5 RNA was treated with TURBO DNaseI (Life Technologies) before reverse transcription with Maxima Reverse Transcriptase (Thermo Fisher Scientific). E7.5 RNA was reverse transcribed using SuperScript III First Strand Synthesis kit (Thermo Fisher Scientific). qRT-PCR was then performed as described above.

### Fluorescence *in situ* Hybridization (FISH)

Fluorescent Cyanine3 (Enzo Life Sciences) and Fluorescein (eEnzyme) probes were made using a Nick Translation Kit (Roche) and column purified (GE Healthcare). RNA FISH, DNA FISH, and combined RNA-DNA FISH were performed as described in [6,40,41]. For each procedure, cells were cytospun onto slides and fixed in 4% paraformaldehyde. RNA FISH: probes incubated with cells on slides for 16 hrs at 37°C, and FISH images were collected on a Zeiss LSM 700 or LSM 780 confocal microscope and analyzed with Volocity software (PerkinElmer); the cell positions for each RNA FISH image were recorded so that the same cells were imaged for the sequential DNA FISH. DNA FISH: cells on slides were treated with RNase A to degrade RNA, followed by treatment with 70% formamide 2XSSC at 80°C to denature DNA; probes hybridized 16 hrs at 42°C and DNA FISH images were collected on the same microscope and analyzed with Volocity. Combined RNA-DNA FISH: performed as described previously [6]. Briefly, slides were treated with 70% formamide 2XSSC at 80°C to denature DNA, without any RNase A treatment, followed by probe hybridization for 16 hrs at 42°C. Combined RNA-DNA FISH images were collected on a Nikon Eclipse 90i microscope and analyzed with Volocity.

### Statistical analyses

Binomial test: compare transgenic and wildtype mouse viability; Female vs. Male and TG vs. WT outcomes are expected at equal ratios based on the breeding scheme WT/WT x TG/WT. Paired student *t*-test: compare female and male average viability ratios. One tailed, unpaired student *t*-test: compare WT and TG expression levels; analysis performed on average expression of ≥2 mEF littermates (N. animals in each category, see Fig 2E); Standard error of the mean is also displayed in Figs 2D, 5B, 5C, S3D and S3E. Chi-square test: compare number of cells with and without *Jpx* or *Xist* RNA clouds in transgenic and wildtype mEFs.

### Reagent and data availability

Transgenic mouse strains are available upon request.

## Supporting information

S1 Fig Ectopic *Xist* expression in male and female mESCs transfected with Tg(Jpx, Xist). (A) Quantitative analysis for *Xist* clouds in female Tg(Jpx, Xist) mESC line #7 as shown Fig 1C, and male Tg(Jpx, Xist) mESC lines #5 and #9 as shown in S1E and S1F Fig. Charts include corresponding *P* values derived from a chi-square test to determine the difference between cloud counts in wildtype and transgenic cells at each differentiation day. (B) Combined RNA-DNA FISH for control mESCs at differentiation days 0, 2, 4, and 8. Female (top) and male (bottom) mESCs were transfected with a Tg(pSKYneo+) control plasmid. Probes used: *Jpx*+*Xist* (green, FITC) and *Xpct* (red, Cy3), as shown in Fig 1A. (C) Combined RNA-DNA FISH for female Tg(Jpx, Xist) mESCs Line #2, at differentiation days 0, 4, and 8. Probes are as indicated in (B). Open arrowhead: Tg(Jpx, Xist) transgenic site. (D) Combined RNA-DNA FISH for female Tg(Jpx, Xist) mESCs Line #11 at differentiation day 8. Probes are as indicated in (B). Open arrowhead: Tg(Jpx, Xist) transgenic site. (E-F) Sequential RNA and DNA FISH on male Tg(Jpx, Xist) mESCs Line #5 (E) and Line #9 (F) at differentiation day 2. RNA FISH probe: *Xist* (green, FITC), DNA FISH probes: *Xist* (green, FITC) and *Xpct* (red, Cy3), as shown in Fig 1A. Open arrowhead: Tg(Jpx, Xist) transgenic site. Scale bar: 2μm. All Tg(Jpx, Xist) mESC lines are stable transgenic cells with single-copy Tg(Jpx, Xist) transgene integrated in an autosome.
(TIF)

S2 Fig *Xist* is expressed from both endogenous and transgenic sites in female and male mEFs. (A) qRT-PCR control reactions for *Gapdh*, *Jpx*, and *Xist* amplification and Ct values obtained with/without reverse transcriptase enzyme in E13.5 Tg(Jpx) transgenic male mEFs. (B) Number of mEFs included in the FISH analysis for Tg(Jpx) lines as shown in Fig 3, and Tg(Jpx, Xist) lines as shown in Fig 4. *P* value is from a chi-square test comparing the RNA cloud counts between wildtype and transgenic samples in each line. (C, D) Diagram of *Xist* expression patterns observed from transgenic Tg(Jpx, Xist) female (C) and male (D) mEFs. The percentage of observed clouds in each category is listed below the diagram. Cells are counted from two transgenic Tg(Jpx, Xist) lines: 04.2 and 05.2.
(TIF)

S3 Fig X-linked gene expression in early embryos. (A) Number of E7.5 embryonic cells included in the FISH analysis for Tg(Jpx) and Tg(Jpx, Xist) as shown in Fig 6. *P* value is from a chi-square test comparing the RNA cloud counts between wildtype and transgenic samples in each line. (B) Number of embryos obtained as littermates and used for the expression analysis. (C) Map of X-Chromosome and genes for quantitative expression analysis in E7.5 embryos. Genes boxed in grey (*Cask*, *Rnf12*, *Atrx*, *Diaph2*) are subject to XCI in mice; genes not boxed (*Kdm6a*, *Eif2s3x*, *Jpx*, *Xist*, *Mid1*) are known to escape XCI in mice. (D) Expression of X-linked genes in E8.5 wildtype and Tg(Jpx) transgenic female embryos from line 95.8. (E) Expression of X-linked genes in E7.5 wildtype and Tg(Jpx) transgenic female and male embryos from line 93.7.
(TIF)

S1 Experimental Procedure List of primers used in the study. (DOCX)

S1 References Additional references for supporting information files. (DOCX)
