# Supplementary figures and images for: LncRNA Jpx induces Xist expression in mice using both trans and cis mechanisms
Source: PLoS Genet. 2018 May 7;14(5):e1007378. doi: 10.1371/journal.pgen.1007378 (PMC5957434; doi:10.1371/journal.pgen.1007378)

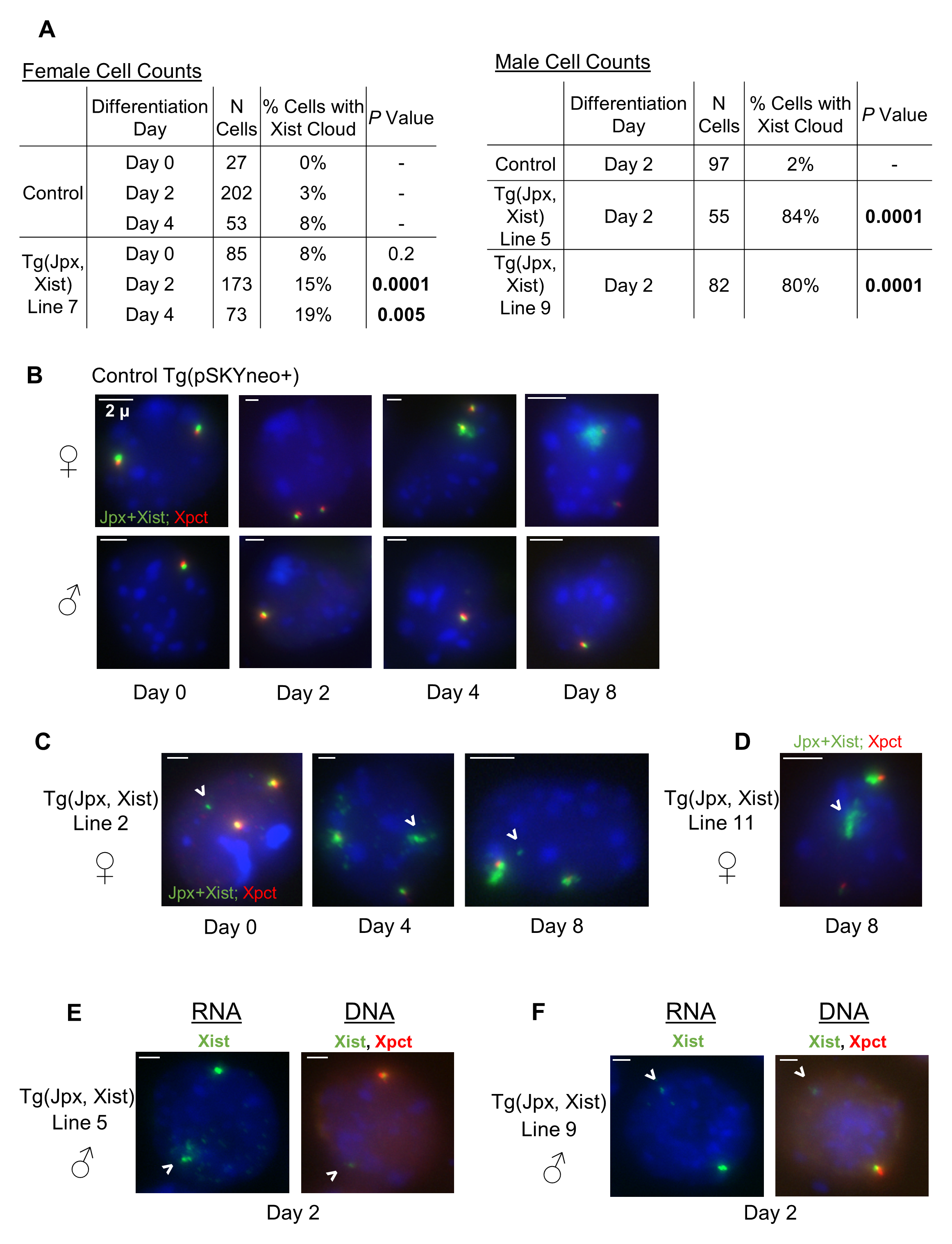

Supplement: S1 Fig — (A) Quantitative analysis for Xist clouds in female Tg(Jpx, Xist) mESC line #7 as shown Fig 1C, and male Tg(Jpx, Xist) mESC lines #5 and #9 as shown in S1E and S1F Fig. Charts include corresponding P values derived from a chi-square test to determine the difference between cloud counts in wildtype and transgenic cells at each differentiation day. (B) Combined RNA-DNA FISH for control mESCs at differentiation days 0, 2, 4, and 8. Female (top) and male (bottom) mESCs were transfected with a Tg(pSKYneo+) control plasmid. Probes used: Jpx+Xist (green, FITC) and Xpct (red, Cy3), as shown in Fig 1A. (C) Combined RNA-DNA FISH for female Tg(Jpx, Xist) mESCs Line #2, at differentiation days 0, 4, and 8. Probes are as indicated in (B). Open arrowhead: Tg(Jpx, Xist) transgenic site. (D) Combined RNA-DNA FISH for female Tg(Jpx, Xist) mESCs Line #11 at differentiation day 8. Probes are as indicated in (B). Open arrowhead: Tg(Jpx, Xist) transgenic site. (E-F) Sequential RNA and DNA FISH on male Tg(Jpx, Xist) mESCs Line #5 (E) and Line #9 (F) at differentiation day 2. RNA FISH probe: Xist (green, FITC), DNA FISH probes: Xist (green, FITC) and Xpct (red, Cy3), as shown in Fig 1A. Open arrowhead: Tg(Jpx, Xist) transgenic site. Scale bar: 2μm. All Tg(Jpx, Xist) mESC lines are stable transgenic cells with single-copy Tg(Jpx, Xist) transgene integrated in an autosome. (TIF) [file pgen.1007378.s001.tif]

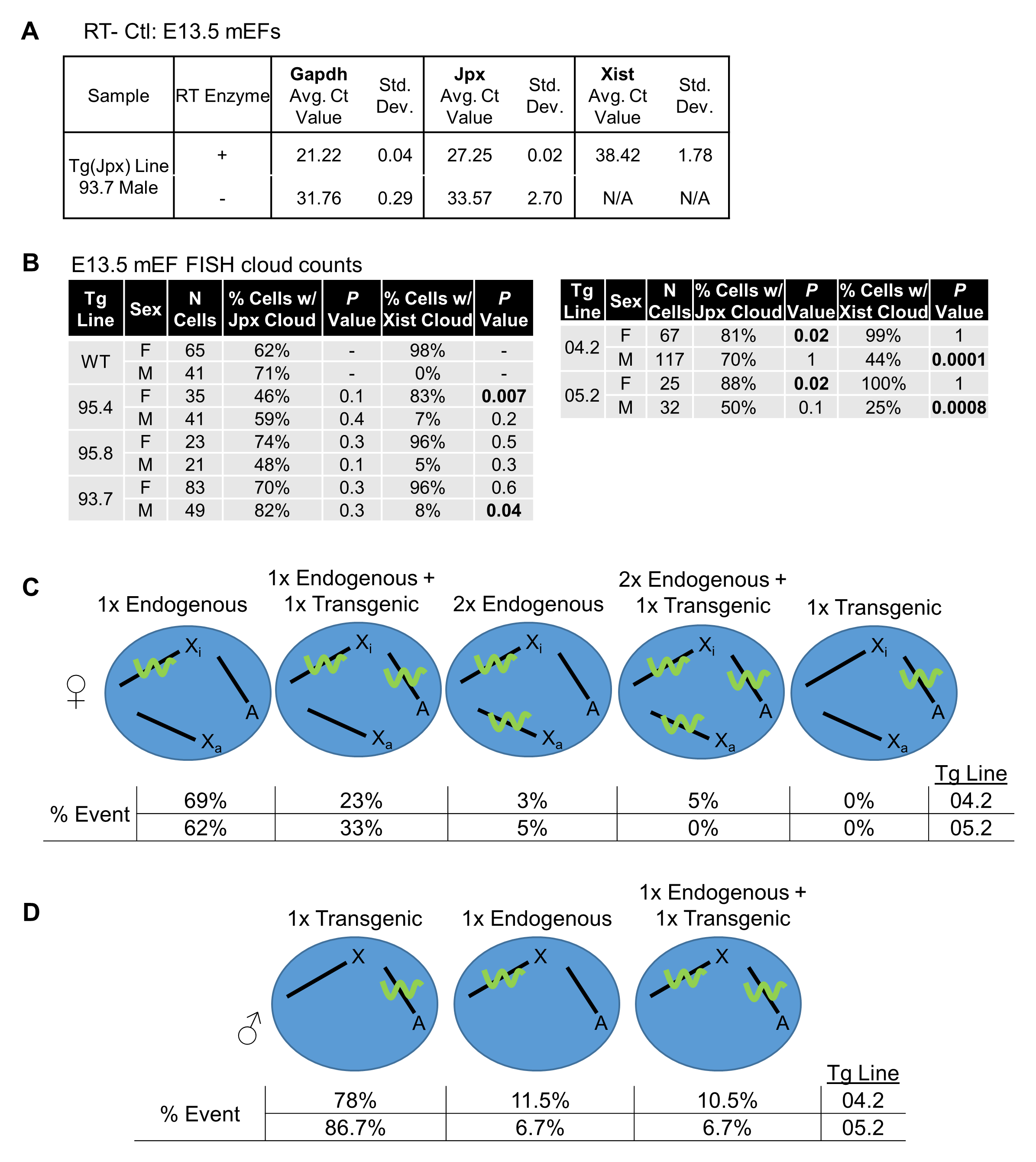

Supplement: S2 Fig — (A) qRT-PCR control reactions for Gapdh, Jpx, and Xist amplification and Ct values obtained with/without reverse transcriptase enzyme in E13.5 Tg(Jpx) transgenic male mEFs. (B) Number of mEFs included in the FISH analysis for Tg(Jpx) lines as shown in Fig 3, and Tg(Jpx, Xist) lines as shown in Fig 4. P value is from a chi-square test comparing the RNA cloud counts between wildtype and transgenic samples in each line. (C, D) Diagram of Xist expression patterns observed from transgenic Tg(Jpx, Xist) female (C) and male (D) mEFs. The percentage of observed clouds in each category is listed below the diagram. Cells are counted from two transgenic Tg(Jpx, Xist) lines: 04.2 and 05.2. (TIF) [file pgen.1007378.s002.tif]

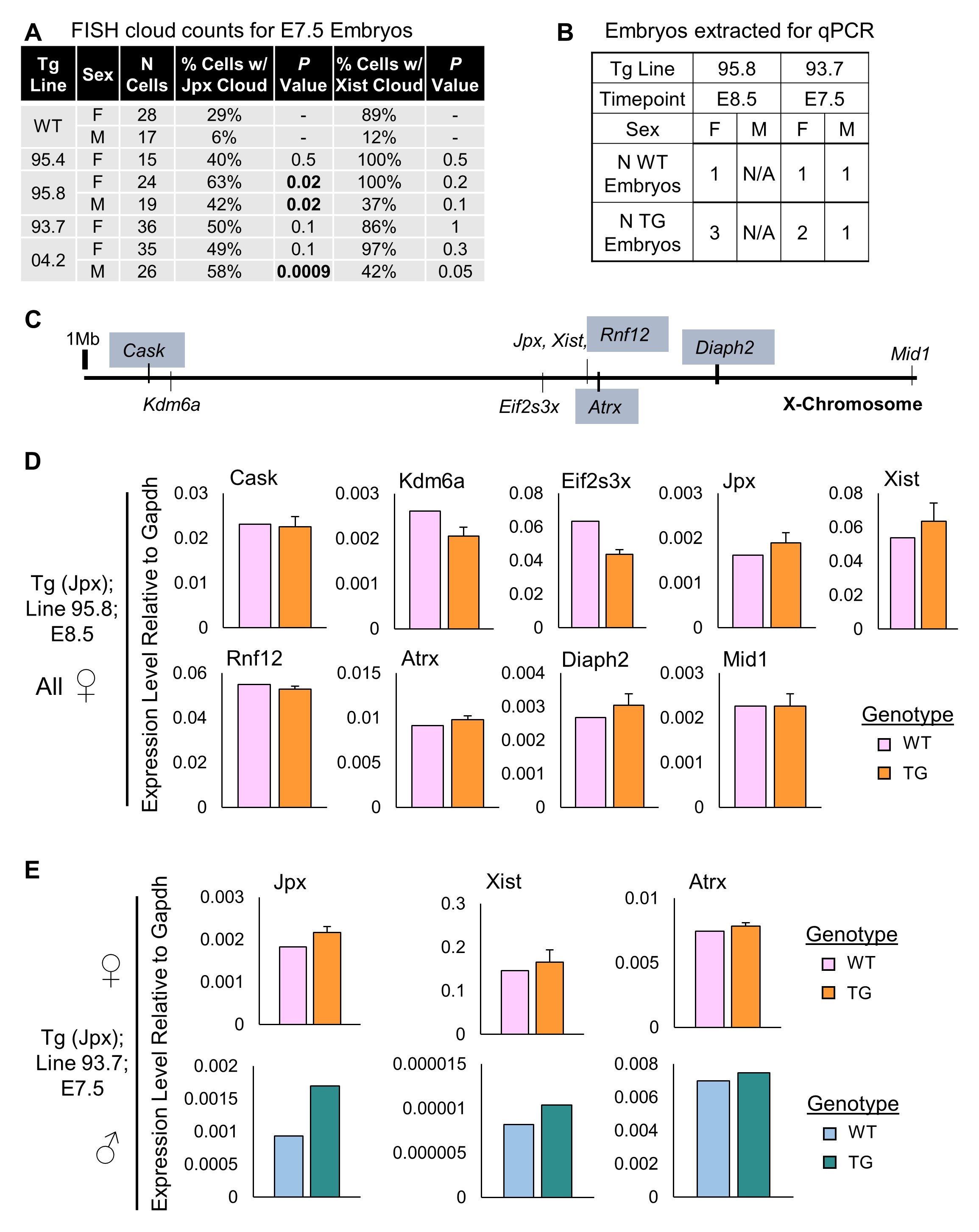

Supplement: S3 Fig — (A) Number of E7.5 embryonic cells included in the FISH analysis for Tg(Jpx) and Tg(Jpx, Xist) as shown in Fig 6. P value is from a chi-square test comparing the RNA cloud counts between wildtype and transgenic samples in each line. (B) Number of embryos obtained as littermates and used for the expression analysis. (C) Map of X-Chromosome and genes for quantitative expression analysis in E7.5 embryos. Genes boxed in grey (Cask, Rnf12, Atrx, Diaph2) are subject to XCI in mice; genes not boxed (Kdm6a, Eif2s3x, Jpx, Xist, Mid1) are known to escape XCI in mice. (D) Expression of X-linked genes in E8.5 wildtype and Tg(Jpx) transgenic female embryos from line 95.8. (E) Expression of X-linked genes in E7.5 wildtype and Tg(Jpx) transgenic female and male embryos from line 93.7. (TIF) [file pgen.1007378.s003.tif]
