# Supplementary material for: LncRNA Jpx induces Xist expression in mice using both trans and cis mechanisms
Source: PLoS Genet. 2018 May 7;14(5):e1007378. doi: 10.1371/journal.pgen.1007378 (PMC5957434; doi:10.1371/journal.pgen.1007378)
Supplement: S1 Experimental Procedure — (DOCX) [file pgen.1007378.s004.docx]

**Supporting Information**

**S1 Experimental Procedure. List of primers used in the study.**

| Procedure/ target | Primer name | Direction | Primer Sequence | Reference |
| --- | --- | --- | --- | --- |
| BAC8 subcloning | BAC8_68199 | F | GGTTTAGGCTCCATTCTTAAGACCTCAT | (Augui et al., 2007; Sun et al., 2015) |
|  | BAC8_68407 | R | GGTCTACAGAGCTAGTTCCAAGACACCA |  |
| BAC8 subcloning | BAC8_147016 | F | ATCCAGGACACCAGGATTCTCC | (Augui et al., 2007; Sun et al., 2015) |
|  | BAC8_147200 | R | CATCTAGAAACACTAGCTTGAGAGG |  |
| Identifying transgenic mice | pBAC4 | F | AGTTGGAACCTCTTACGTGCCGAT | (Sun et al., 2015) |
|  |  | R | ATGTGGTGTGACCGGAACAGAGAA |  |
| Confirming sex of mice | UBEX/Y | F | TGGTCTGGACCCAAACGCTGTCCACA | (Senner et al., 2011) |
|  |  | R | GGCAGCAGCCATCACATAATCCAGATG |  |
| Jpx gene copy number | JpxEx1 | F | GCA CCA CCA GGC TTC TGT AAC | (Tian et al., 2010) |
|  |  | R | GGG CAT GTT CAT TAA TTG GCC AG |  |
| Xist gene copy number | XistP2Y | F | CTC GAC AGC CCA ATC TTT GTT | (Jeon and Lee, 2011) |
|  | XistP2C | R | ACC AAC ACT TCC ACT TAG CC |  |
| Hprt gene (X-Chr.) | Hprt_12848 | F | CTG CTA CTT CAA CTC CTG GTG TGC | (Sun et al., 2015) |
|  | Hprt_12970 | R | AGG CGA ATT GGG ATG TAG CTC AG |  |
| Jpx transcript | mJpx76+ (Exon 1) | F | TTAGCCAGGCAGCTAGAGGA | (Sun et al., 2013) |
|  | mJpx225- (Exon 2) | R | AGCCGTATTCCTCCATGGTT |  |
| Xist transcript | XistBP2 | F | CCCGCTGCTGAGTGTTTGATA | (Payer et al., 2013) |
|  | XistNS33 | R | CAGAGTAGCGAGGACTTGAAGAG | (Stavropoulos et al., 2001) |
| Gapdh transcript | GapdhBP1 | F | ATG AAT ACG GCT ACA GCA ACA GG | (Payer et al., 2013) |
|  |  | R | CTC TTG CTC AGT GTC CTT GCT G |  |
| Mid1 | Mid1FY | F | AGCCTGTGGAGTCCATCAAC | (Yang et al., 2010) |
|  |  | R | GCTTTCAGGCACTCATCACA |  |
| Diaph2 | Diaph2_2943F | F | AAGCGCAGGCAAAGTTTCAG | This manuscript |
|  | Diaph2_3203R | R | TCCATGTTTACTGTGTTCGGGT |  |
| Atrx | Atrx_264F | F | AGCCCATGAGTGGAAACAAGT | This manuscript |
|  | Atrx_368R | R | CAAGTCGTGGAGAAGAACACG |  |
| Rnf12 | Rnf12_518F | F | TAAAGAGGGTCCACCACCAC | (Barakat et al., 2014) |
|  | Rnf12_676R | R | GCTCTCCAGGACTGGTTTCC | This manuscript |
| Eif2s3x | EifFY | F | CTTTATCAGGGGCAGAGCAG | (Yang et al., 2010) |
|  |  | R | AGCCTCAGACACCCAGTGTT |  |
| Kdm6a | Kdm6a_4617F | F | ATCAACATGCTCCTCCATTACCA | This manuscript |
|  | Kdm6a_4845R | R | GCTTTACGAGAGTCCTGGCA |  |
| Cask | Cask_2456F | F | TGGTGGCAGGGTAAACTGGA | This manuscript |
|  | Cask_2698R | R | TGCTGGCAGTTTGACTACTTCT |  |
