# Supplementary material for: LncRNA Jpx induces Xist expression in mice using both trans and cis mechanisms
Source: PLoS Genet. 2018 May 7;14(5):e1007378. doi: 10.1371/journal.pgen.1007378 (PMC5957434; doi:10.1371/journal.pgen.1007378)
Supplement: S1 References — (DOCX) [file pgen.1007378.s005.docx]

**Supporting Information**

**S1 References**

Augui, S., Filion, G.J., Huart, S., Nora, E., Guggiari, M., Maresca, M., Stewart, A.F., and Heard, E. (2007). Sensing X Chromosome Pairs Before X Inactivation via a Novel X-Pairing Region of the Xic. Science (80-. ). *318*, 1632–1637.

Barakat, T.S., Loos, F., Van Staveren, S., Myronova, E., Ghazvini, M., Grootegoed, J.A., and Gribnau, J. (2014). The trans-activator RNF12 and cis-acting elements effectuate X chromosome inactivation independent of X-pairing. Mol. Cell *53*, 965–978.

Jeon, Y., and Lee, J.T. (2011). YY1 Tethers Xist RNA to the inactive X nucleation center. Cell *146*, 119–133.

Payer, B., Rosenberg, M., Yamaji, M., Yabuta, Y., Koyanagi-Aoi, M., Hayashi, K., Yamanaka, S., Saitou, M., and Lee, J.T. (2013). Tsix RNA and the germline factor, PRDM14, link X reactivation and stem cell reprogramming. Mol. Cell *52*, 805–818.

Senner, C.E., Nesterova, T.B., Norton, S., Dewchand, H., Godwin, J., Mak, W., and Brockdorff, N. (2011). Disruption of a conserved region of Xist exon 1 impairs Xist RNA localisation and X-linked gene silencing during random and imprinted X chromosome inactivation. Development *138*, 1541–1550.

Stavropoulos, N., Lu, N., and Lee, J.T. (2001). A functional role for Tsix transcription in blocking Xist RNA accumulation but not in X-chromosome choice. Proc. Natl. Acad. Sci. U. S. A. *98*, 10232–10237.

Sun, S., Del Rosario, B.C., Szanto, A., Ogawa, Y., Jeon, Y., and Lee, J.T. (2013). Jpx RNA Activates Xist by Evicting CTCF. Cell *153*, 1537–1551.

Sun, S., Payer, B., Namekawa, S., An, J.Y., Press, W., Catalan-Dibene, J., Sunwoo, H., and Lee, J.T. (2015). Xist imprinting is promoted by the hemizygous (unpaired) state in the male germ line. Proc. Natl. Acad. Sci. *112*, 14415–14422.

Tian, D., Sun, S., and Lee, J.T. (2010). The long noncoding RNA, Jpx, is a molecular switch for X-chromosome inactivation. Cell *143*, 390–403.

Yang, F., Babak, T., Shendure, J., and Disteche, C.M. (2010). Global survey of escape from X inactivation by RNA-sequencing in mouse. Genome Res. *20*, 614–622.
